# Astrocytic and Oligodendrocytic P2X7 Receptors Determine Neuronal Functions in the CNS

**DOI:** 10.3389/fnmol.2021.641570

**Published:** 2021-02-09

**Authors:** Ya-Fei Zhao, Yong Tang, Peter Illes

**Affiliations:** ^1^School of Acupuncture and Tuina, Chengdu University of Traditional Chinese Medicine, Chengdu, China; ^2^International Collaborative Center on Big Science Plan for Purine Signaling, Chengdu University of Traditional Chinese Medicine, Chengdu, China; ^3^Rudolf Boehm Institute for Pharmacology and Toxicology, University of Leipzig, Leipzig, Germany

**Keywords:** P2X7 receptors, astrocytes, oligodendrocytes, neurons, signaling molecules

## Abstract

P2X7 receptors are members of the ATP-gated cationic channel family with a preferential localization at the microglial cells, the resident macrophages of the brain. However, these receptors are also present at neuroglia (astrocytes, oligodendrocytes) although at a considerably lower density. They mediate necrosis/apoptosis by the release of pro-inflammatory cytokines/chemokines, reactive oxygen species (ROS) as well as the excitotoxic (glio)transmitters glutamate and ATP. Besides mediating cell damage i.e., superimposed upon chronic neurodegenerative processes in Alzheimer’s Disease, Parkinson’s Disease, multiple sclerosis, and amyotrophic lateral sclerosis, they may also participate in neuroglial signaling to neurons under conditions of high ATP concentrations during any other form of neuroinflammation/neurodegeneration. It is a pertinent open question whether P2X7Rs are localized on neurons, or whether only neuroglia/microglia possess this receptor-type causing indirect effects by releasing the above-mentioned signaling molecules. We suggest as based on molecular biology and functional evidence that neurons are devoid of P2X7Rs although the existence of neuronal P2X7Rs cannot be excluded with absolute certainty.

## Introduction

As a last member of the ATP-gated cationic channel family P2X, the P2X7 receptor (R) was cloned and characterized by Surprenant et al. (1996). These authors already described several important properties of the receptor, such as stimulation by pathophysiologically large concentrations of extracellular ATP in the high micromolar range, a gradual increase of the receptor-current, when induced by repetitive or continuous activation, and a unique long cytoplasmic tail which was required for the lytic/necrotic actions of ATP. An increase of P2X7R current amplitude on long-lasting contact with ATP was thought for many years to be due to progressive dilation of the P2X7R channel (Virginio et al., 1999). Dilation of the receptor-channel itself or its association with the membrane channel pannexin-1 (Panx-1; Pelegrin and Surprenant, 2006) was supposed to allow the entry of large molecules (e.g., Yo-Pro-1) into the cell interior or the loss of important cell ingredients into the extracellular space leading to necrosis. However, more recently convincing evidence has been presented that these earlier hypotheses are probably misleading (Di Virgilio et al., 2018; Ugur and Ugur, 2019) and that the P2X7R channel permits the passage of large cationic molecules immediately from its initial activation, but at a much slower pace than that of the small cations Na^+^, K^+^, and Ca^2+^ (Harkat et al., 2017). Further, it has been proposed that the C-terminus of the P2X7R is needed for activation of the macropore and, in consequence for membrane permeabilization (Smart et al., 2003). However, it appears that the lack of a C-terminal tail does not fully abolish, but only slows down the formation of the macropore (Karasawa et al., 2017).

There has been a long-lasting controversy whether P2X7Rs are localized at neurons, or whether neuronal effects are indirect, due to the stimulation of astrocytic P2X7Rs, which on their behalf interact with neurons *via* releasing various signaling molecules (Illes et al., 2017; Miras-Portugal et al., 2017). This overview aims to discuss arguments supporting the idea that neuroactive P2X7Rs are probably localized at astrocytes/oligodendrocytes, and enlist novel molecular biology results suggesting that these receptors are missing at neurons. We did not intend to give a relatively complete survey of the relevant literature as presented in our recent review (Illes et al., 2017), but rather limited ourselves to the most relevant aspects of the neuroglia-mediated signaling to neurons.

## Astrocyte-Neuron Interaction *Via* P2x7 Receptors

### Astrocytic P2X7Rs

In the CNS, P2X7Rs occur at the highest density at microglia (Bhattacharya and Biber, 2016; Illes et al., 2020), but are present also at astrocytes as confirmed by mRNA and protein measurements (Fischer et al., 2009; Illes et al., 2012). Hippocampal (Kukley et al., 2001) as well as accumbal and cortical astrocytes of rats stained for P2X7R immunoreactivity (IR; Franke et al., 2012), although the latter ones only after mechanical or ischemic injury. Similarly, cerebral cortices of AD patients and an AD mouse model (APPPS1) expressed P2X7R-IR in astrocytes (Martin et al., 2019).

For some time, it was questioned whether the immunohistochemically identified astrocytic P2X7Rs have also functional significance (Jabs et al., 2007), but later unequivocal evidence was forwarded to confirm this assumption (Illes et al., 2012; Verkhratsky et al., 2012). ATP and the prototypic P2X7R agonist dibenzoyl-ATP (Bz-ATP) induced current responses in mixed astrocyte/neuron cultures prepared from the rodent neocortex (Duan et al., 2003; Nörenberg et al., 2010; Rubini et al., 2014) and in brain slices of the pre-frontal cortex (Oliveira et al., 2011). The measurement of the ATP-induced increase of intracellular Ca^2+^ ([Ca^2+^]_i_) also confirmed the existence of functional P2X7Rs at cultured astrocytes (Fumagalli et al., 2003; Fischer et al., 2009).

### Astrocytic Signaling *via* P2X7R Activation

**Figure 1:**
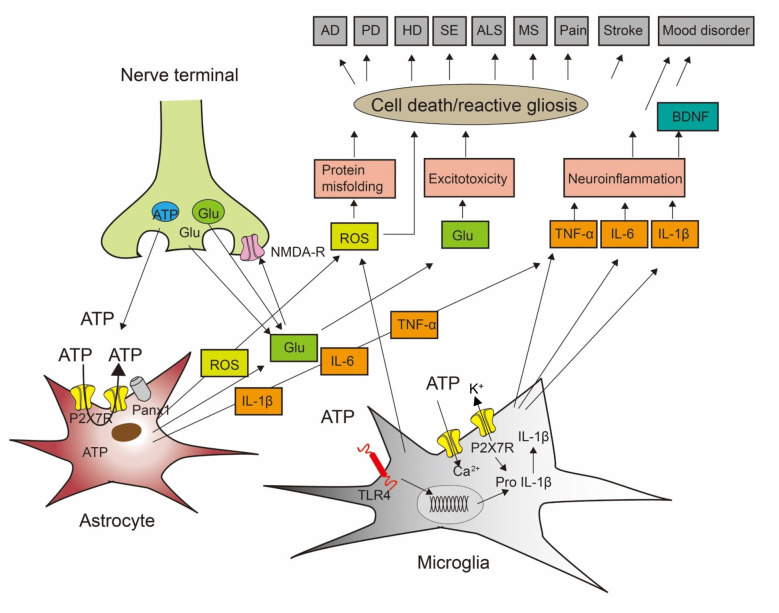
Cross-talk between neurons, astrocytes, and microglia to induce cell death during neurodegenerative and psychiatric illnesses. ATP and glutamate (Glu) are neurotransmitters released from nerve terminals but also gliotransmitters released/secreted from astrocytes and microglia. In consequence, ATP may be released by exocytotic mechanisms from neurons/astrocytes, but also through the P2X7 receptors (P2X7Rs) themselves or pannexin-1 (Panx-1) channels from astrocytes. P2X7Rs are nonselective cationic channels and thereby entry pathways for extracellular Ca^2+^ into the cell interior and exit pathways for the loss of K^+^. Stimulation of the toll-like receptor TLR4R by lipopolysaccharide (LPS) primes the generation of the inflammatory cytokine interleukin-1β (IL-1β) by the cleavage of pro-IL-1β. This process involves nucleotide-binding, leucine-rich repeat, pyrin domain containing 3 (NLRP3) inflammasome-mediated caspase-1 activation. Impoverishment in cytoplasmic K^+^ by P2X7R activation is a major stimulus for NLRP3 activation and therefore boosts IL-1β production and secretion. Presynaptic *N*-methyl-D-aspartate (NMDA) receptors facilitate the release of glutamate. Reactive oxygen species (ROS) are secreted from microglial cells. ROS induces protein misfolding, glutamate is in high concentrations an excitotoxin, and the inflammatory cytokines IL-1β, IL-6, and tumor necrosis factor-α (TNF-α) cause neuroinflammation, leading also to the release of brain-derived neurotrophic factor (BDNF). AD, Alzheimer’s disease; PD, Parkinson’s disease; HD, Huntington’s disease; SE, status epilepticus; ALS, amyotrophic lateral sclerosis; MS, multiple sclerosis. Modified with permission from Illes et al. (2019b).

Astrocytes may communicate with neurons using several signaling molecules released by exocytotic and non-exocytotic pathways (Figure 1; Illes et al., 2019a). In the first line, the P2X7R pore is believed to be involved in the production and release of neuroinflammatory cytokines. The best characterized immune response associated with P2X7R stimulation is a leucine-rich repeat, pyrin domain containing 3 (NLRP3) inflammasome activation and the consequent interleukin-1β (IL-1β) secretion from macrophages/microglia (Di Virgilio et al., 2017; Illes et al., 2020). The maturation and release of IL-1β and IL-18 are mediated by NLRP3 stimulation, but activated microglia may secrete also further pro-inflammatory cytokines such as IL-6 and tumor necrosis factor-α (TNF-α) by other mechanisms (Shieh et al., 2014). Although the primary source of pro-inflammatory cytokines are microglial cells, astrocytes also appear to participate in this process and release IL-1β, IL-6, and TNF-α (Choi et al., 2014).

Reactive oxygen species (ROS) are another signaling molecule released by macrophages/microglia *via* P2X7R-induced activation of NADPH oxidase 2 (Apolloni et al., 2014; Bartlett et al., 2014). Astrocytes were also described to increase ROS production through NADPH oxidase, subsequently leading to IL-6 production (Munoz et al., 2020). The above-mentioned class of cytokines and chemokines, such as CCL2 or CCL5, as well as ROS, are neuroinflammatory molecules putting a further burden onto neurons already damaged by various neurodegenerative illnesses such as Alzheimer’s disease (AD; Illes et al., 2019b), Parkinson’s disease (PD; Carmo et al., 2014), amyotrophic lateral sclerosis (Apolloni et al., 2014), and multiple sclerosis (MS; Domercq and Matute, 2019). However, this damage is mostly due to a massive microglial outflow of cytokines/chemokines, while their astrocytic counterparts are likely to induce more moderate reactions such as depressive-like behavior in rodents and major depression in humans by interacting with cortico-limbic neuronal circuits (Koo and Duman, 2009; Maes et al., 2012).

### Astrocytic Glutamate/GABA Stimulate Neighboring Neurons

In addition to these pro-inflammatory mediators the activation of astrocytic P2X7Rs was shown to release the (glio)transmitters glutamate (Duan et al., 2003; Bardoni et al., 2010), GABA (Wang et al., 2002; Wirkner et al., 2005), and ATP (Suadicani et al., 2006; Xiong et al., 2018). The potent, although non-selective P2X7R agonist Bz-ATP induced transient and tonic glutamate release, manifesting as transient (slow inward current; SIC) and tonic current, respectively in CA1 neurons of hippocampal slices (Fellin et al., 2006). The SICs were due to *N*-Methyl-D-Aspartate (NMDA) receptor activation (blocked by AP5), while the tonic current responses were mediated by P2X7Rs (blocked by oxidized ATP). In dorsal horn lamina II (substantia gelatinosa, SG) neurons of spinal cord slices, only SICs were recorded after Bz-ATP application (Bardoni et al., 2010). Both in the hippocampus and in the spinal cord, the source of glutamate exerting the respective electrophysiological responses were supposed to be astrocytes, which exhibited [Ca^2+^]_i_ elevations accompanying the transmembrane currents.

In good correlation with the above findings, Bz-ATP evoked inward currents in CA1 and lamina II neurons and astrocytes kept in slice preparations; a low Ca^2+^/no Mg^2+^ (low X^2+^)-containing incubation medium was used in both cases to facilitate the P2X7R-mediated responses (Ficker et al., 2014; Gao et al., 2017). Whereas the antagonism of P2X7Rs strongly inhibited both the neuronal and astrocytic current responses, a combination of antagonists for AMPA, NMDA and GABA_A_Rs almost abolished the Bz-ATP-induced current amplitudes in the neurons only (Ficker et al., 2014). It was concluded that P2X7Rs are localized at astrocytes, the stimulation of which causes the release of glutamate (and probably GABA) stimulating their respective receptors at the neighboring neurons. Also, Bz-ATP potentiated the frequency of spontaneous postsynaptic currents in CA1 neurons (Khan et al., 2019). This effect on CA1 neurons was inhibited by the GABA_A_R antagonist gabazine or the astrocytic toxin fluorocitrate, suggesting stimulation of P2X7Rs at stratum radiatum astrocytes synapsing onto CA1 neurons. A stimulatory effect of astrocytic P2X7Rs executed *via* the release of glutamate onto noradrenergic locus coeruleus neurons was recorded as an increased frequency of the glutamatergic spontaneous excitatory postsynaptic currents (sEPSCs) of these neurons (Khakpay et al., 2010).

### Astrocytic ATP Stimulates Neighboring Neurons

Similar to glutamate/GABA also ATP itself may be released from astrocytes *via* P2X7R stimulation to modulate neuronal functions. As mentioned in the Introduction, Panx-1 channels are opened by activation of P2X7Rs (Pelegrin and Surprenant, 2006; Locovei et al., 2007), and both ATP release and dye uptake induced by this stimulus are blocked by Panx-1 knockdown strategies (Iglesias et al., 2008; Suadicani et al., 2012). In the rat hypothalamic paraventricular nucleus, an increase in the amplitude of miniature excitatory postsynaptic currents (mEPSCs) was observed in response to noradrenaline that released ATP from glial cells (Gordon et al., 2005). It was suggested that ATP acts at postsynaptic P2X7Rs localized at magnocellular neurosecretory cells to promote the insertion of AMPA receptors, resulting in the up-regulation of synaptic efficacy (i.e., mEPSC amplitude).

Whereas pannexin channels only function as release pathways from astrocytes, two opposed connexins form gap junctions between individual astrocytes connecting the cytoplasm of adjacent cells (Verkhratsky and Nedergaard, 2018; Theis and Giaume, 2012). Gap junctions serve as pathways for the exchange of small molecules of less than about 1 kDa, including ions, energy metabolites, neurotransmitters, and signaling molecules, that co-ordinate the metabolic and functional activities of connected cells (Pannasch and Rouach, 2013; Cheung et al., 2014). On the other hand, unopposed connexin hemichannels are exit pathways for small molecules from the intracellular space allowing e.g., the release of ATP in a similar manner as pannexins do. It is noteworthy, that although the excitotoxic glutamate/ATP were the primary gliotransmitters released from astrocytes, GABA may be also released under certain conditions to mediate inhibition of neuronal circuits.

However, glutamate, GABA, ATP, and the glutamatergic co-agonist D-serine may be also outpoured from astrocytes by Ca^2+^-dependent, exocytotic/vesicular mechanisms (Lalo et al., 2014a, b). They were suggested to be released from astrocytic processes enwrapping the contact points (synapses) of the presynaptic neuronal elements with the dendritic specializations of the postsynaptic neuron. This tri-partite synapse is ideally suited to serve as an astrocyte-neuron communication interface (Araque et al., 1999; Halassa and Haydon, 2010).

## Oligodendrocyte-Neuron Interaction *Via* P2x7 Receptors

### Neurotoxic P2X7Rs at Oligodendrocytes

Although astrocytes in the CNS have attracted a lot of attention, oligodendrocytes outnumber them by almost a factor of two and are certainly of comparable importance for neuronal functions (Pelvig et al., 2008; Illes et al., 2019a). They constitute the myelin sheath of neuronal fiber tracts and thereby insulate individual axons permitting spatially separated and rapid conduction of action potentials. Ample evidence documents that oligodendrocytes and their precursors express P2X7Rs (Matute, 2008); Bz-ATP evoked an elevation in the cytosolic concentration Ca^2+^, and the cationic dye Yo-Pro-1 was taken up by oligodendrocytes in optic nerve glia (James and Butt, 2002; Matute, 2008; Butt, 2011). P2X7Rs in oligodendrocytes are highly permeable to Ca^2+^ and prolonged activation of these receptors is lethal to differentiated oligodendrocytes in culture and to mature oligodendrocytes in isolated optic nerves *in vitro* and *in vivo* (Matute, 2008). In this respect, ATP would act like the excitotoxic glutamate and damage the white matter of the CNS (Matute, 2011).

Primary cultures of oligodendrocytes derived from optic nerves of rats were subjected to *in vitro* ischemia achieved by replacing O_2_ with N_2_, external glucose with sucrose, and adding iodoacetate to the incubation medium to block glycolysis (Domercq et al., 2010). This treatment induced inward current, which could be reversed by the P2X7R antagonist Brilliant Blue G (BBG), the ATP degrading enzyme apyrase, and by blockers of pannexin hemichannels. It was suggested that ischemia releases ATP from cultured oligodendrocytes, which activates P2X7Rs at these very cells aggravating ischemic damage. Furthermore, sustained activation of P2X7Rs *in vivo* causes morphological lesions that are reminiscent of the major features of MS plaques, i.e., demyelination, oligodendrocyte death, and axonal damage (Matute et al., 2007). In an animal model of MS, experimental autoimmune encephalomyelitis, treatment with BBG ameliorated the neurological symptoms and restored normal axon conduction velocity in the optic nerve.

### Possible Involvement of Oligodendrocytic P2X7Rs in Developmental Effects

As early as in 2006 it has been suggested that axonal firing stimulates different purinergic receptors at Schwann cells and oligodendrocyte progenitors regulating development and myelination (Fields, 2006). Moreover, P2X7Rs were found to evoke Ca^2+^ transients in growth cones of e.g., axons of hippocampal neurons kept in culture and thereby interfere with axonal growth (Diaz-Hernandez et al., 2008). Pharmacological inhibition of P2X7Rs or its silencing by shRNA interference induced longer and more branched axons, coupled with morphological changes in the growth cone. Signaling by this receptor was through adenylate cyclase which also coordinated its interaction with P2Y1 and P2Y13Rs which promoted axonal elongation (del Puerto et al., 2012). It is well established that adult neural progenitor cells (NPCs) located in the so-called neurogenic niches of the brain and being the common ancestors of newly generated neurons, astrocytes, and oligodendrocytes possess P2X7Rs (Tang and Illes, 2017; Leeson et al., 2019). Hence, we tentatively suggest that growth cones are developmentally immature cellular constituents that express NPC-like properties regulating axonal growth and branching.

The axon initial segment is the site where action potentials are triggered due to the high concentration of voltage-gated ion channels (Bender and Trussell, 2012). In dissociated cultured hippocampal neurons and hippocampal slice cultures, P2X7R activation decreased both sodium current amplitude and intrinsic neuronal excitability while P2X7R inhibition had the opposite effect (Del Puerto et al., 2015). Although not explicitly stated by the authors, we assume that P2X7Rs were situated at oligodendrocytes rather than the neurons themselves, implicating the signaling by oligodendrocyte cell products to neurons. Of course, the participation of astrocytes cannot be excluded either.

### Oligodendrocyte Precursor Cells (NG2 Glia)

Besides astrocytes and oligodendrocytes, NG2 glial cells (expressing the proteoglycan NG2 in addition to the classic immunohistochemical markers for oligodendrocytes) emerged during the last decades as the third type of neuroglial cells in the CNS (Seifert and Steinhäuser, 2018; Bedner et al., 2020). These cells are also referred to as oligodendrocyte precursor cells (OPCs) because in the white matter they differentiate into myelinating oligodendrocytes (Nishiyama et al., 2016). However, in the gray matter, NG2 glia does not differentiate into oligodendrocytes but maintains its phenotype throughout postnatal life. These phenotypic characteristics include the expression of functional voltage-gated K^+^, Na^+^, and Ca^2+^ channels, without the ability to generate action potentials (Haberlandt et al., 2011). Rodent NG2 glia receives direct synaptic inputs from glutamatergic and GABAergic neurons, a feature that is unique among glial cells (Bergles et al., 2000). It is noteworthy that in the hippocampus, the number of NG2 cells amounts to about 20–25% of that of astrocytes (Degen et al., 2012).

We suggest that P2X7R possessing NG2 cells should be also considered as oligodendrocyte-like executors of neuronal effects. ATP/Bz-ATP increased [Ca^2+^]_i_ in purified (less than 2% astrocytes and microglia), cultured NG2-immunopositive OPCs (Agresti et al., 2005). The Bz-ATP-induced raise in [Ca^2+^]_i_ fully depended on extracellular Ca^2+^ and was blocked in presence of the P2X7R antagonistic oxidized ATP.

Thus, there is strong, direct evidence for the presence of neurotoxic P2X7Rs at oligodendrocytes and less convincing, circumstantial evidence for developmentally active P2X7Rs at the same cell type. However, at the moment it is difficult to judge whether astrocytic or oligodendrocytic (NG2 glia) P2X7Rs are more important for signaling to neurons (see below current molecular biology evidence for the presence of this receptor at oligodendrocytes but not astrocytes).

## Neuronal P2X7 Receptors *per se*?

Numerous experimental reports are suggesting assumedly unequivocal evidence for neuronal P2X7Rs. However, we will prove below for a few prominent examples that this evidence allows also the conclusion that P2X7Rs are localized at astrocytes/oligodendrocytes which by their signaling molecules indirectly modify neuronal functions.

### Electrophysiological Evidence in Cell Culture, Brain Slice, and Isolated Cell Preparations

In segments of the guinea-pig ileum, myenteric neurons were impaled by sharp microelectrodes or patch-clamp pipettes, and ATP or Bz-ATP was used to evoke inward currents (Hu et al., 2001; Valdez-Morales et al., 2011). These currents were abolished by the P2X7R antagonistic oxidized ATP and Brilliant Blue G. However, all these preparations contained an ample population of glial cells certainly expressing P2X7Rs.

More refined methods were also used to elucidate the question of neuronal P2X7Rs. Immature hilar neurons with attached glutamate releasing nerve terminals were mechanically isolated from rat hippocampal slices (Cho et al., 2010). Bz-ATP increased the frequency of sEPSCs, and this effect was blocked by Brilliant Blue G. In most of the hilar neurons tested, the Bz-ATP-induced increase in sEPSC frequency was blocked by tetrodotoxin or Cd^2+^, suggesting that the activation of P2X7Rs leads to a presynaptic depolarization.

In an ingenious methodological approach, mechanical deformation of isolated retinal ganglion cells led to the release of ATP through pannexin hemichannels which were supposed to act back directly at the neurons to auto-stimulate their P2X7Rs (Xia et al., 2012). P2X7R activation was measured as an A438079 (selective P2X7R antagonist)-sensitive cationic inward current. Ganglion cells were seeded onto elastic silicone material and were introduced into a cell stretch chamber; all figures of the paper show data generated on a mixed retinal cell preparation, meaning the additional presence of astrocytes/Müller cells. In some cases, the neurobasal medium (NBM) was used for culture to obtain purified neuronal preparations. This procedure was however found to cause the acquirement of astrocytic properties by cultured striatal neurons (Rubini et al., 2006). These neurons kept in NBM fired only a single low amplitude spike on injection of a depolarizing current pulse, while their counterparts that were grown in Dulbecco’s modified Eagle’s medium (DMEM) fired series of high amplitude action potentials. Also, neurons cultured in NBM responded to ATP with [Ca^2+^]_i_ transients due to the release of Ca^2+^ from intracellular pools, otherwise a property of only astrocytes in DMEM grown cultures.

### Presynaptic P2X7Rs

It has repeatedly been reported that when hippocampal brain slices or cultured cerebrocortical cells were pre-incubated with [^3^H]glutamate or [^3^H]GABA, ATP/Bz-ATP caused a release of the transmitters previously incorporated into their storage pools. All effects were blocked by conventional P2X7R antagonistic drugs (Sperlagh et al., 2002; Wirkner et al., 2005) or by using P2X7R knockout (KO) mice (Papp et al., 2004).

More convincing evidence for the existence of presynaptic P2X7Rs was supplied, when pinched-off nerve terminals (synaptosomes) were prepared from various parts of the rodent and human brain (mouse cerebellum, Sanchez-Nogueiro et al., 2005; rat caudal brainstem, D’Amico et al., 2010; Curro et al., 2020; neocortex of epileptic humans, Barros-Barbosa et al., 2018). Once again, after loading of the transmitter pools with radioactively labeled excitatory amino acids, subsequent application of ATP/Bz-ATP led to the release of tritium considered to be a measure of transmitter release.

P2X7R stimulation can initiate the influx of Ca^2+^ through the membrane of mice midbrain synaptosomes (Marin-Garcia et al., 2008). This was documented by microfluorimetric measurements of [Ca^2+^]_i_ transients induced by ATP/Bz-ATP and the blockade of this effect by Brilliant Blue G.

All these results were considered to be convincing evidence for the exclusive neuronal location of release-regulating P2X7Rs. However, it was not taken into account that the crude synaptosomal preparations used are not really homogenous and contain also glial material, microsomes, and mitochondria (Henn et al., 1976; Olde and Johansson, 1989).

### Retinal Ganglion Cells

P2X7R-mRNA and protein have been identified in the rat retina at different postnatal developmental stages (Brändle et al., 1998). In the human retina, P2X7Rs are present in Müller cells, where they function as targets of ATP, coupled with Ca^2+^-mediated signaling pathways (Pannicke et al., 2000). In preparations obtained from patients suffering from proliferative vitreoretinopathy, stimulation by Bz-ATP of human Müller cells induced larger membrane currents than in preparations from healthy patients (Bringmann et al., 2001).

Genetic association studies have implicated purinergic receptors in the development of age-related macular degeneration (Gu et al., 2013). It was suggested that P2X7Rs identified by their immunoreactivity at retinal ganglion cells may be the reason for the observed apoptotic reactions (Franke et al., 2005). A similar approach was used to explain the deleterious molecular changes initiated by P2X7R activation in the mice glaucomatous retina (Perez de Lara et al., 2019). However, the allegedly specific increase in P2X7R-IR and function of neurons is tightly accompanied by similar changes in retinal glial cells probably being the primary targets of large extracellular ATP concentrations.

### Stress-Induced Increase in P2X7Rs

It has repeatedly been suggested that neuronal P2X7Rs may be absent or down-regulated at resting conditions but become expressed on stressful conditions such as temporal lobe epilepsy (Engel et al., 2012; Jimenez-Pacheco et al., 2016), ischemic damage to the brain (Franke et al., 2004), neuropathic pain (Andó and Sperlagh, 2013; Zhang et al., 2019), and neurodegenerative diseases (AD; Diaz-Hernandez et al., 2012; Rodrigues et al., 2015; Huntington’s disease and PD, Diaz-Hernandez et al., 2009; Glaser et al., 2020; MS, Sharp et al., 2008; Grygorowicz et al., 2010). Without any doubt these injurious conditions activate microglial cells as well as astrocytes/oligodendrocytes and facilitate the release of neurodamaging signaling molecules (cytokines, chemokines, reactive oxygen and nitrogen species, glutamate/ATP), possibly facilitating neuronal functions. However, the more marked and most likely spurious immunohistochemical staining of P2X7Rs at neurons is by no way an indication of their existence as functional receptors.

### Developmental Models for P2X7Rs

Adult NPCs of the subgranular zone in the mouse hippocampus contain functional P2X7Rs, while in the mature granule cells, the developmental successors of the NPCs, P2X7Rs are missing (Rozmer et al., 2017). Undifferentiated Neuro-2a neuroblastoma cells possess functional P2X7Rs as proven by [Ca^2+^]_i_ transients and inward current responses caused by Bz-ATP application (Gomez-Villafuertes et al., 2009). Neuronal differentiation of these cells by retinoic acid treatment was associated with decreases in the expression and function of P2X7Rs (Wu et al., 2009). Inhibition of P2X7Rs by pharmacological antagonists or shRNA interference resulted in increased neurogenesis, whereas P2X7R overexpression significantly reduced the formation of neurites (Gomez-Villafuertes et al., 2009; Wu et al., 2009). These data as a whole suggest that during neuronal differentiation of neuroglia-like cells the P2X7R function successively decreases.

In the above paragraphs, we enumerated a few experimental findings purportedly supporting the existence of neuronal P2X7Rs. However, because of the obvious difficulties in obtaining completely glia-free neuronal preparations, considerable doubts remain whether P2X7Rs are situated at the neurons themselves. We have to concede that our criticism does not exclude the possibility of neuronal P2X7Rs but together with the molecular biology evidence summarized in the next section, strongly argues for the absence of this receptor type at neurons.

## Knockout and Transgenic P2x7 Receptor Mice Models

### Knockout Strategies

The above results acquire significance because of a hypothesis forwarded recently on the non-neuronal localization of P2X7Rs (Rubini et al., 2014; Illes et al., 2017; Khan et al., 2019). It was suggested that glial, especially astrocytic P2X7Rs are indirect causes of neuronal effects. Initial doubts concerning the existence of neuronal P2X7Rs were raised by the observation that polyclonal antibodies binding to this receptor-type stained not only hippocampal structures of wild-type mice but also those of two separate P2X7R KO strains generated by the pharmaceutical companies Pfizer or Glaxo Smith Kline (GSK; Sim et al., 2004). This apparent riddle was resolved some time afterward, by demonstrating that none of the two KO strategies eliminated all immunologically and functionally active splice variants of the genuine receptor P2X7A (Bartlett et al., 2014; Sperlagh and Illes, 2014). In the GSK mouse, a widely expressed and strongly functional P2X7k splice variant escaped inactivation (Nicke et al., 2009). In the Pfizer mouse, C-terminally truncated variants of P2X7A (ΔC) were not depleted; when expressed in HEK293 cells, this variant inefficiently trafficked to the cell surface and agonist-evoked currents were small (Masin et al., 2012). Co-expressed with P2X7A, ΔC-P2X7 acted in a dominant-negative fashion to depress current responses.

More recently, a humanized *P2rx7* allele generated by Metzger et al. (2017) was accessible to spatially and temporally controlled Cre-recombinase-mediated inactivation. In contrast to the “classic” KO mice, none of the described *P2rx7* splice variants evaded this null allele. By selective disruption and assessment of *P2rx7* expression in different brain regions and cell types it was found to exist in astrocytes, oligodendrocytes, and microglia, as repeatedly shown by P2X7-protein measurements (see previous sections), but only in a single neuronal structure of the brain, the glutamatergic pyramidal neurons of the hippocampal CA3 region. It is still unproven that this very circumscriptive expression of mRNA has an appropriate protein readout.

A powerful technique to investigate the cell-type-specific distribution of *P2rx7* was for a couple of years the bacterial artificial chromosome (BAC)-transgenic reporter mouse model Tg(P2rx7-EGFP) FY174 Gsat^1^, a sequence encoding soluble enhanced green fluorescent protein (sEGFP) under the control of the *P2rx7* promoter. Experimental results obtained on this mouse model were considered to strongly support the existence of neuronal P2X7Rs in the brain (Garcia-Huerta et al., 2012; Miras-Portugal et al., 2017; Martinez-Frailes et al., 2019). Specifically, status epilepticus induced by kainic acid infusion into the nucleus amygdala demonstrated the neuronal expression and up-regulation of P2X7R-mRNA in the hippocampus (Engel et al., 2012; Jimenez-Pacheco et al., 2016).

### Transgenic Strategies

However, later on, the Tg(RP24-114E20P2X7451P-StrepHis-EGFP)Ani reporter mouse was generated (Kaczmarek-Hajek et al., 2018). Extensive characterization of these transgenic mice revealed dominant P2X7-EGFP protein expression in microglia, Bergmann glia, and oligodendrocytes, but not in neurons. These findings were confirmed by microglia- and oligodendrocyte-specific P2X7 deletion and a novel P2X7-specific nanobody. By using this model mouse, status epilepticus induced by intra-amygdala injection of kainic acid confirmed co-localization of P2X7-EGFP with cell-type-specific markers in oligodendrocytes, but not in neurons or astrocytes (Morgan et al., 2020).

Although in both P2X7 reporter models the EGFP constructs are expected to be expressed under the control of the *P2rx7* promoter, there were substantial differences in their cell-type-specific expression, and serious concerns were raised regarding the reliability of the reporter expression in the sEGFP mouse (Ramirez-Fernandez et al., 2020). For example, the sEGFP model overexpresses a P2X4 passenger gene, and sEGFP shows clear neuronal localization, but appears to be absent in microglia.

## Conclusions

In conclusion, molecular biology and functional data suggest that P2X7Rs are probably missing in neurons of the CNS, while their presence in neuroglial cells (astrocytes, oligodendrocytes) can be taken for granted. Thus, P2X7R-mediated effects in neurons are suggested to be indirect, due to neuroglia-neuron interaction by various signaling molecules. Nonetheless, at the present stage, the existence of neuronal P2X7Rs cannot be negated with absolute certainty.

## Author Contributions

PI drafted the manuscript. All authors contributed to the article and approved the submitted version.

## Conflict of Interest

The authors declare that the research was conducted in the absence of any commercial or financial relationships that could be construed as a potential conflict of interest.

## Footnotes

^1^https://www.gensat.org/
